# Can Long-Term Results Following Balloon Angioplasty Be the “Crystal Ball” to Predict Outcome Following Bioresorbable Vascular Scaffolds?

**DOI:** 10.1161/JAHA.112.005272

**Published:** 2012-10-25

**Authors:** Antonio Colombo, Azeem Latib

**Affiliations:** San Raffaele Scientific Institute and EMO-GVM Centro Cuore Columbus, Milan, Italy

**Keywords:** editorials, bioresorbable vascular scaffold, coronary stent, drug-eluting stent, late stent thrombosis, stent thrombosis

In this issue, Yamaji et al^1^ report the very long-term (15-year) outcomes of patients treated successfully with balloon angioplasty (BA) compared with bare-metal stent implantation (BMS). Considering the major progresses made in interventional cardiology since the advent of BA, it would be reasonable to question what relevance these data have in the era of third- and fourth-generation drug-eluting stents (DESs) that have biodegradable polymers or are polymer free or have a completely bioabsorbable platform. This report comes at an opportune moment in interventional cardiology because of the emergence and availability of the ABSORB everolimus-eluting bioresorbable vascular scaffold (BVS; Abbott Vascular, Santa Clara, CA). These data might be our “crystal ball” into predicting late and very late events after nonpermanent stent alternatives such as drug-eluting balloons (DEBs) or BVS (the BA group) or after implantation of a DES with biodegradable or no polymers (the BMS group).

In the current study, Yamaji et al^1^ report the very long-term outcomes of patients who were free from early restenosis. The main findings were the following:

- The cumulative incidence of all-cause death (44.4% versus 45.4%), cardiac death (19.5% versus 20.6%), or composite of death or myocardial infarction (52% versus 51.6%) at 15 years was similar for the BA and BMS groups
- The cumulative incidence of target lesion revascularization (TLR) was higher in the BA group (44.6% versus 36.0% at 15 years, log-rank *P*<0.001). The difference in TLR rates was most marked within the first year after the index procedure (31.5% versus 16.1% at 14 months), with a plateau phase between 14 months and 4 years (33.8% versus 18.5% at 4 years).
- Late TLR (4 to 15 years) occurred less frequently after BA (16.3% versus 21.4%).
- In the BA group, less residual stenosis at early follow-up angiography was associated with a lower incidence of late TLR.
- Target lesion thrombosis after 1 year was similar for the BA and BMS groups (1.5% versus 0.7%).

In applying these data, we must realize that there are a number of limitations that may make the vision through our crystal ball hazy and limit the scope of our predictions. The authors have clearly outlined these limitations and the large selection bias of the patients in this study, which limits the clinical applicability of the conclusions. Furthermore, regarding generalizability, these data do not apply to patients undergoing percutaneous coronary intervention for acute myocardial infarction or in vessels <3 mm in diameter. Keeping these limitations in mind, let's attempt to use this study to make predictions about late events with current-generation devices.

## Prediction #1. Will late events (in particular, repeat revascularization) in those with a BVS be similar to those in the BA group and thus lower than with a permanent metallic prosthesis?

The available data have confirmed the efficacy of the drug-elution profile of BVSs.^2–5^ The first-generation ABSORB BVS was not associated with repeat revascularization at 4 years,^6^ and the second iteration of ABSORB BVS 1.1 was associated with a 3.6% TLR rate at 12 months.^5^ However, we do not know what plaque progression will be in a scaffolded segment when the artery is no longer caged. Furthermore, after the initial phase of drug elution, we do not know if BVSs will have a plateau phase between 1 and 4 years like the BA and BMS groups in this study or a progressive, albeit slow, increase in TLR as seen with both first- and second-generation DESs with durable polymers.^7^ However, after 4 years, it reasonable to assume that BVSs will have a TLR pattern like that in the BA group. We could even assume that BVSs will perform better, concerning very late events, because of the passivation action of everolimus without the trigger of the foreign body: the metal.

The low very late TLR rate after BA is very encouraging; nevertheless, these findings can be interpreted in 2 ways: (1) the lack of a foreign body, as with a BMS, allows a better “restitutio” of the vascular integrity with all the advantages of unaltered physiology and (2) the BA group is “cleaned” by excluding patients who underwent early TLR (of whom there were more in the BA group than in the BMS group). Indeed, a major limitation of this study with extended follow-up is that early events tend to be more frequent with BA, which results in a selected population of patients. This fact gives the BA group a clear advantage.

The findings reported by these authors can also be used to predict the long-term outcome after a successful angioplasty with a DEB. If this were true, then looking through our crystal ball, the cumulative incidence of TLR after a DEB procedure, given the 6-month TLR rate of 4.4% in the recently completed BELLO (Balloon Elution and Late Loss Optimization) trial,^8^ might be about 21% at 15 years (see Figure 3 in Yamaji et al^1^).

However, we would expect the results with BVSs to be superior to that of the BA group solely on the basis that the BVS will result in a better angiographic result and less residual stenosis. Lower residual stenosis at the end of the procedure and at early follow-up angiography is known to be associated with less TLR during follow-up. Analogously, patients in the present study with a smaller percentage diameter stenosis at early follow-up angiography had a lower late TLR rate than those with a larger diameter stenosis (14.5% versus 28.0%). The average diameter stenosis in this group (26.7±7.1%) is similar to that seen at 6 months with a second-generation BVS (24.0±9.6%).^4^ Furthermore, as experience with postdilating BVS increases, postprocedural and early follow-up diameter stenoses should become even lower, thus resulting in an even lower late TLR rate. Another theoretical advantage of BVSs that could further contribute to lower late TLR is that degradation of struts might remove the ongoing inflammatory stimulus for intimal hyperplasia and the absence of a vessel cage would not prevent late positive remodeling.^3,9^

## Prediction #2. Will the rates of late revascularization with a biodegradable polymer or polymer-free DES be similar to those after BMS implantation?

Although DESs are extremely efficacious in inhibiting intimal hyperplasia while the drug is being eluted, they clearly change the underlying plaque and function of neointima in a way that is different than that of BMSs. Indeed, we do not know if second-/third-generation biodegradable polymer or polymer-free DESs will behave as BMSs after the drug has eluted, with regard to late events and neoatherosclerosis. It is now accepted that neoatherosclerosis is more frequent and occurs significantly earlier in DESs than in BMSs.^10,11^ However, unstable lesions characterized as thin-cap fibroatheromas or plaque rupture are more frequent in BMSs probably because of the longer implant duration. In all cases, however, there is usually no communication between the lesion within the stent and the underlying native atherosclerotic plaque.^11^ In rare cases, neoatherosclerosis may contribute to very late revascularization and thrombotic events in both BMSs and DESs. However, pathological data regarding neoatherosclerosis in DESs are limited to first-generation DESs with durable polymers.

In the LEADERS (Limus Eluted from A Durable versus ERodable Stent coating) trial, the TLR rate at 4 years of a biodegradable polymer DES was 9%.^12^ If we look through our crystal ball again, can we predict that these DESs will behave like BMSs after the polymer has degraded? If so, then these stents will have a late TLR rate of 1.6%/y, like the BMS group in the present study (see Figure 3 in Yamaji et al^1^). However, the cumulative incidence curves will probably be very different and continue to progress slowly without showing a plateau phase. It is not known if this is related to neoatherosclerosis or just a more protracted neointimal response than occurs with BMSs.

## Prediction #3. Will very late target lesion thrombotic events be lower with biodegradable or polymer-free DESs and BVSs compared with BMSs or BA, respectively?

The causes of late stent thrombosis have been described as related to delayed endothelialization, chronic inflammatory response, and localized hypersensitivity reactions.^9,13^ All these adverse reactions can be potentially prevented by the implantation of a fully biodegradable stent.^9,13^ Similarly, in the LEADERS trial, the 4-year rates of very late definite stent thrombosis were significantly lower with biodegradable polymer DESs than with durable polymer DESs (0.4% versus 1.8%; *P*=0.004).^12^

The most important and challenging prediction to make is whether BVSs remove the risk of very late stent thrombosis from the clinical arena. To predict whether late events after implantation of a BVS will be similar to those after a BA procedure, we need to know if the mechanisms of healing and late events are similar. In this regard, an intriguing and important finding in the present study is that target lesion thrombosis was similar in the 2 groups.^1^ Indeed, late target lesion thrombosis (after 4 years) was present in 7 of 10 patients with BA who underwent late TLR for acute myocardial infarction. Does this suggest that the absence of a permanent vascular scaffold does not protect from late thrombotic events? This would be a very controversial statement as the enthusiasm for BVSs is based on the fact that after degradation, the treated segment of the vessel will return to normal function, thus eliminating the dependence on long-term dual antiplatelet therapy and the risk of late thrombotic events.^14^ This concept is also circumstantially supported by data demonstrating normal vasomotion after BVS implantation.^3–5,14,15^

However, there is a paucity of long-term histopathological data after BA or BVS. It is not known if late events are due to further expansion of the underlying plaque or rupture/erosion of neoatherosclerotic plaques. One hypothesis is that BVSs may seal plaque, resulting in more “normal healing” and thus altering neoatherosclerosis. Brugaletta et al^16^ provided some preliminary data in this direction with their observation that BVSs result in a symmetrical and circumferential thick fibrous cap. The premise is that this healing process of BVSs might be used to stabilize vulnerable plaques before the scaffold disappears and leaves the vessel uncaged.^17^

In conclusion, BA and current stenting do not seem to fully protect the vessel from neoatherosclerosis or plaque progression. It may be possible that a fully bioresorbable scaffold, by eliminating the presence of a permanent vascular prosthesis, may passivate the vessel, thus resulting in better healing and a vessel less prone to atherosclerosis. Presently, there are insufficient scientific and clinical data to support the dream that sealing or passivation of critical and selected noncritical coronary plaques with nonpermanent scaffolds may prevent future events.
